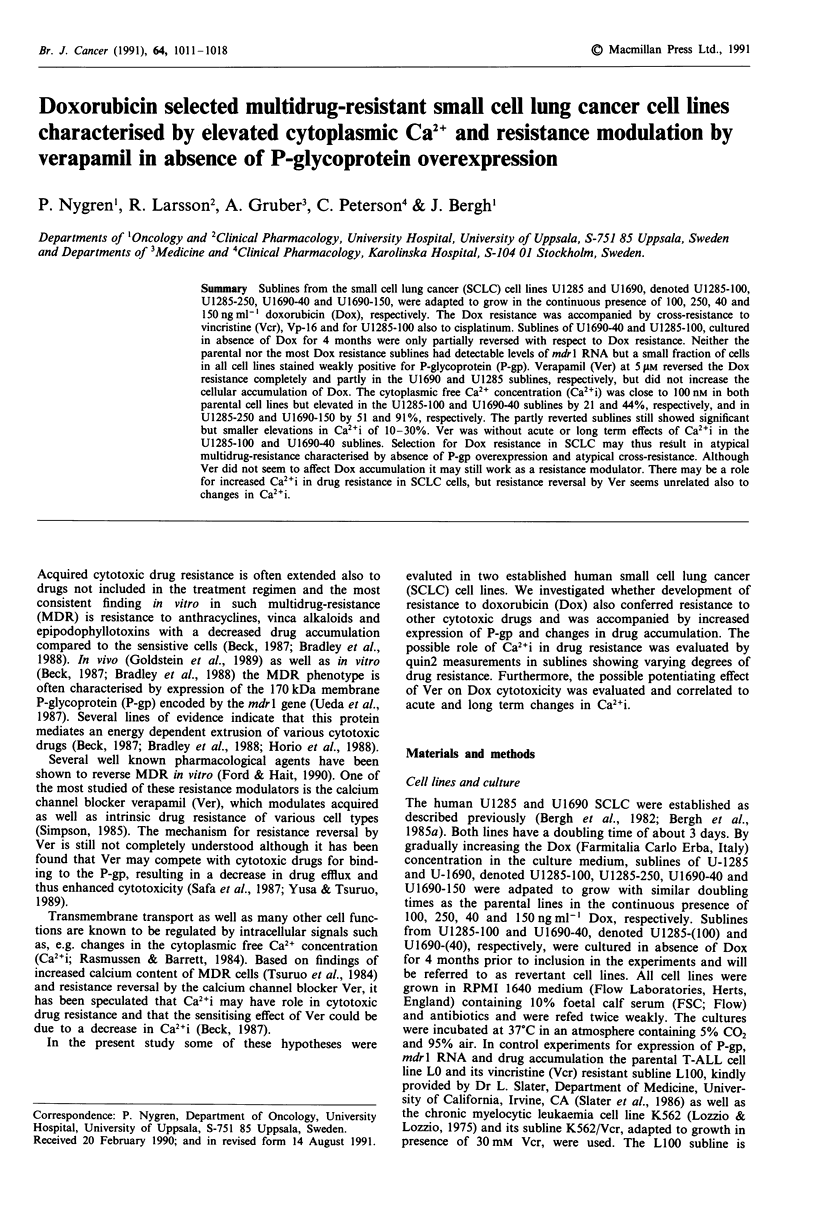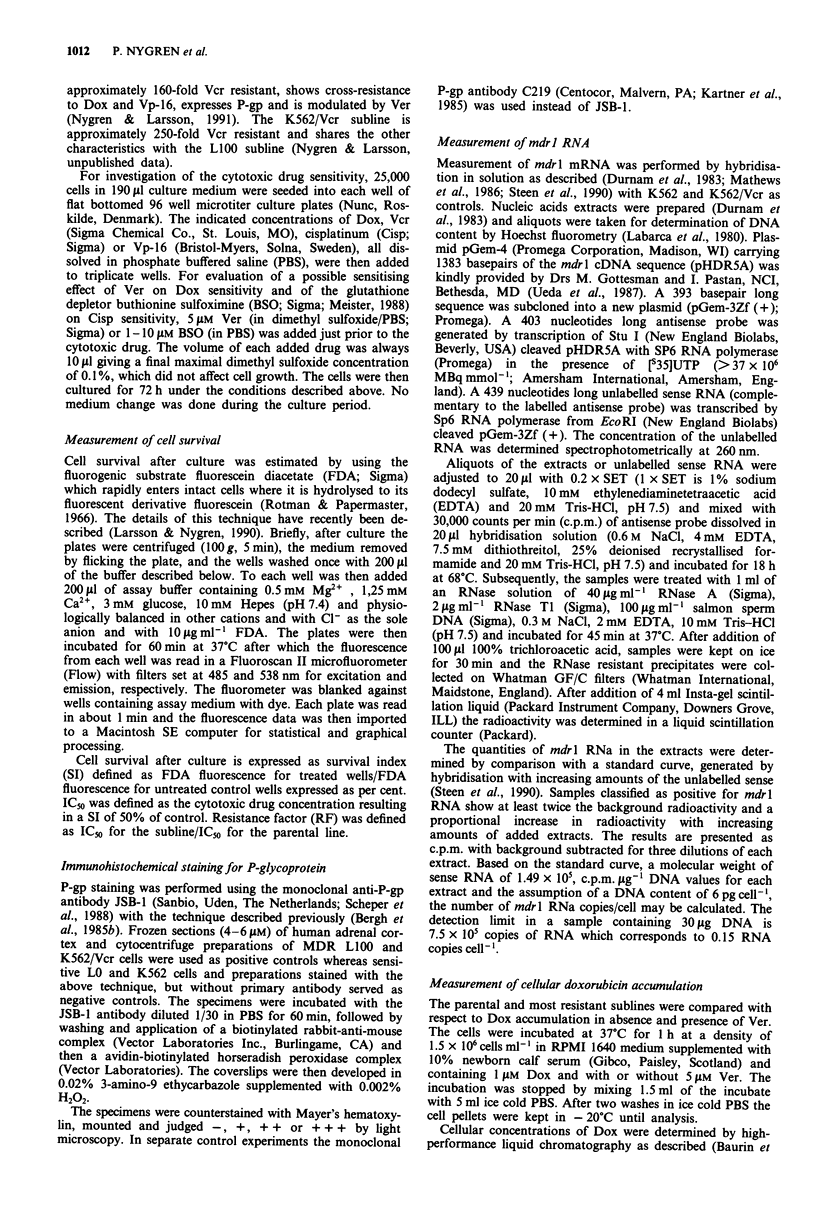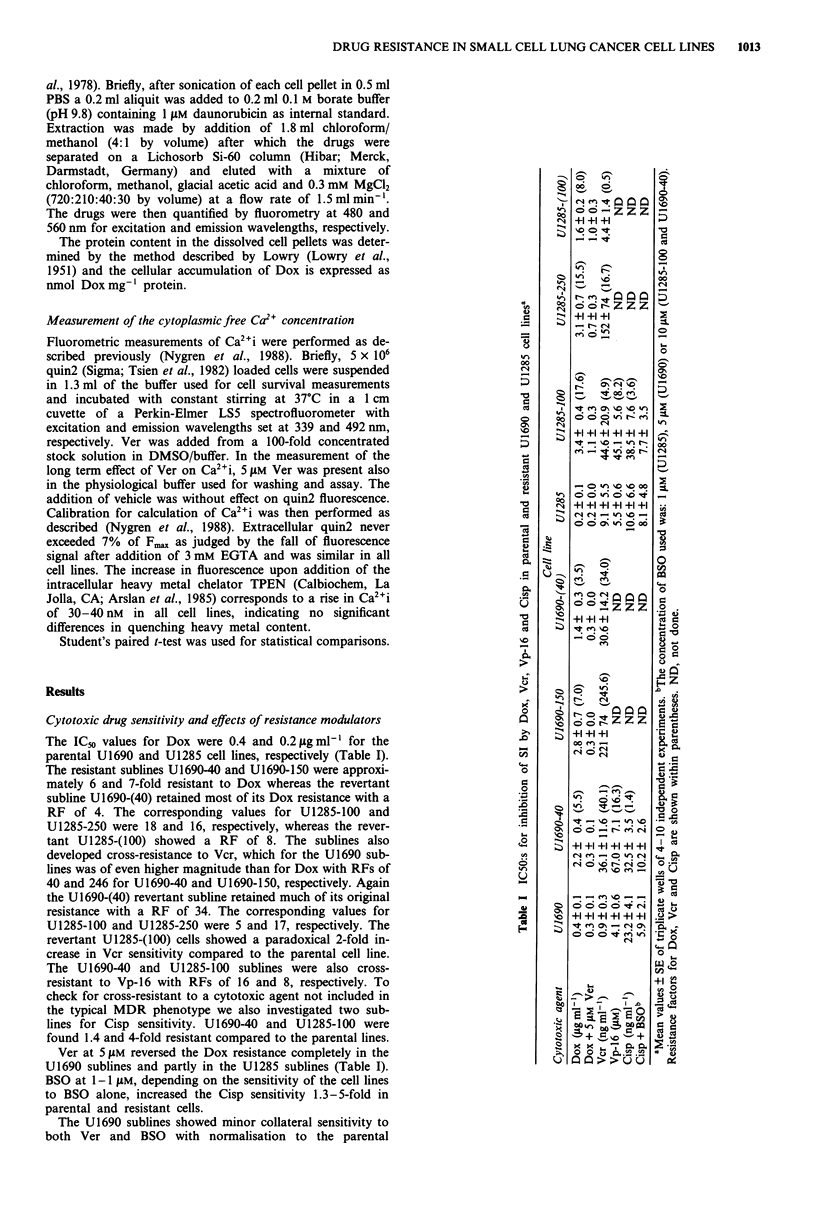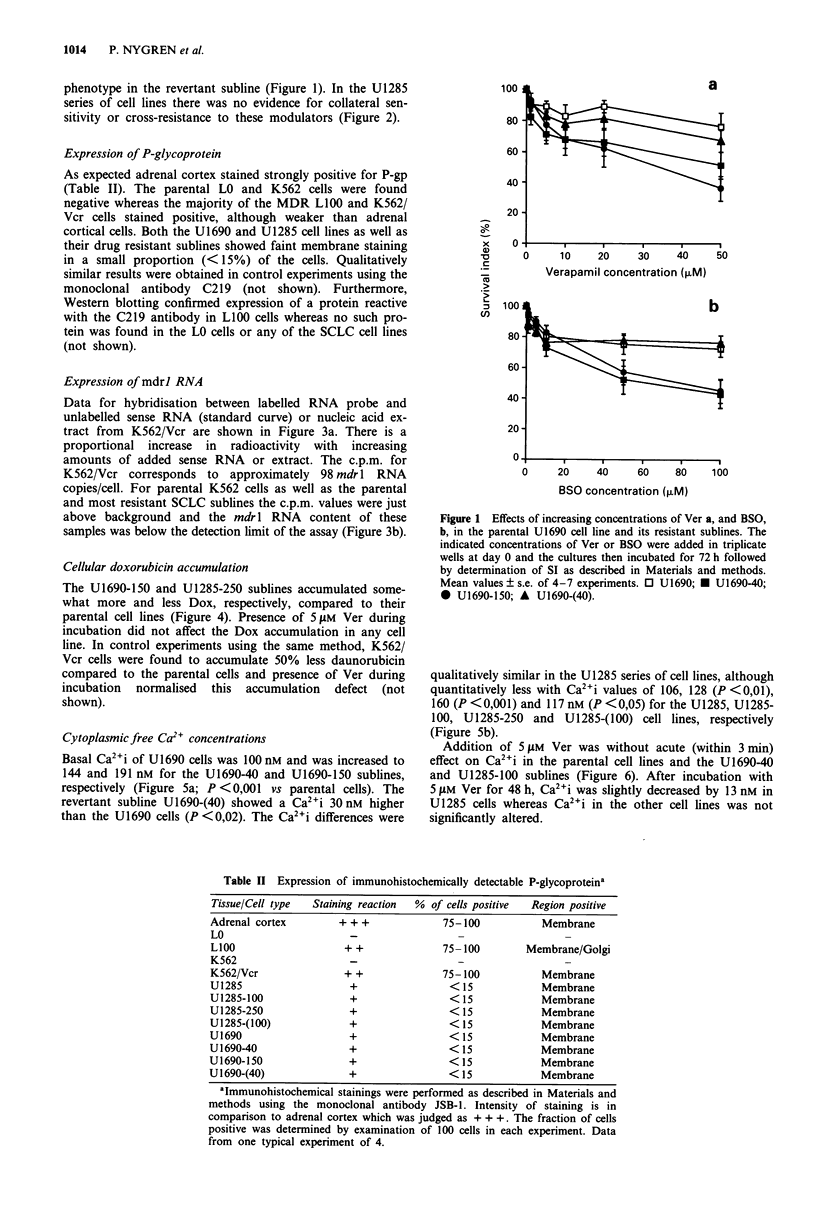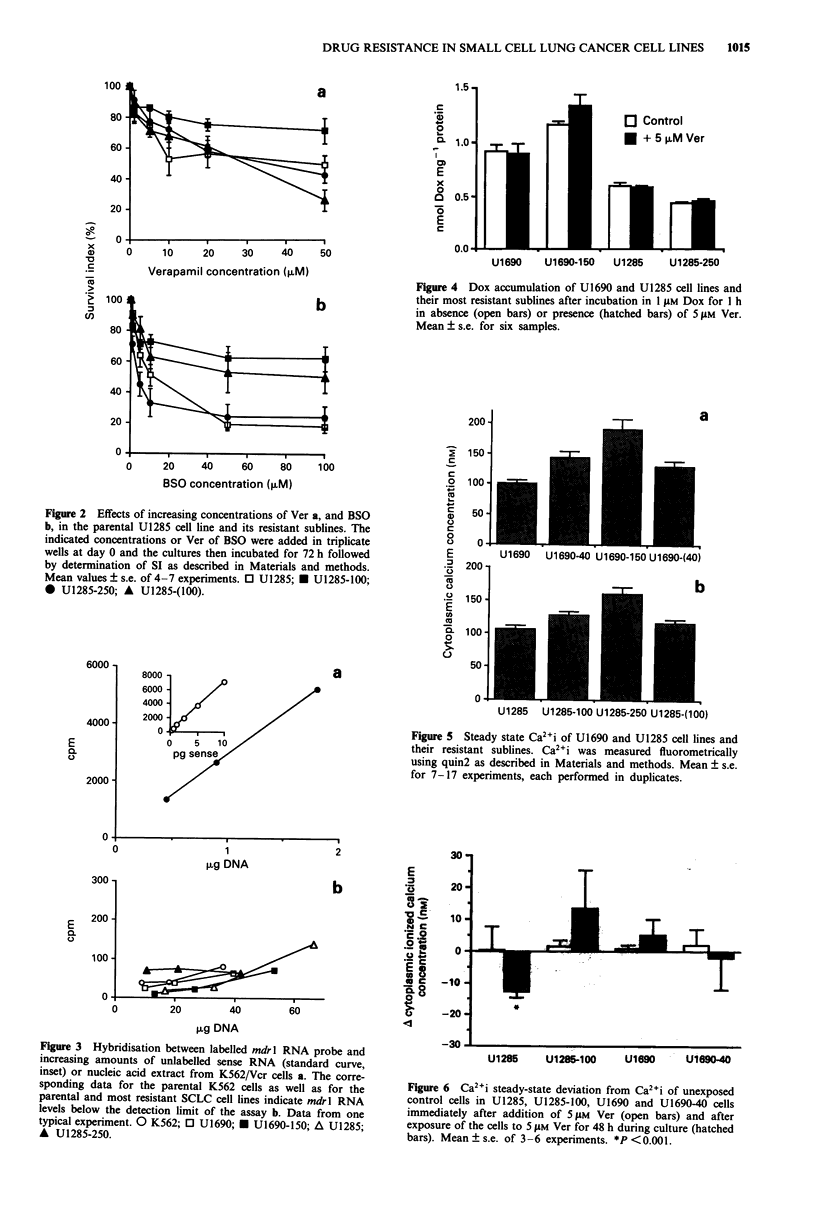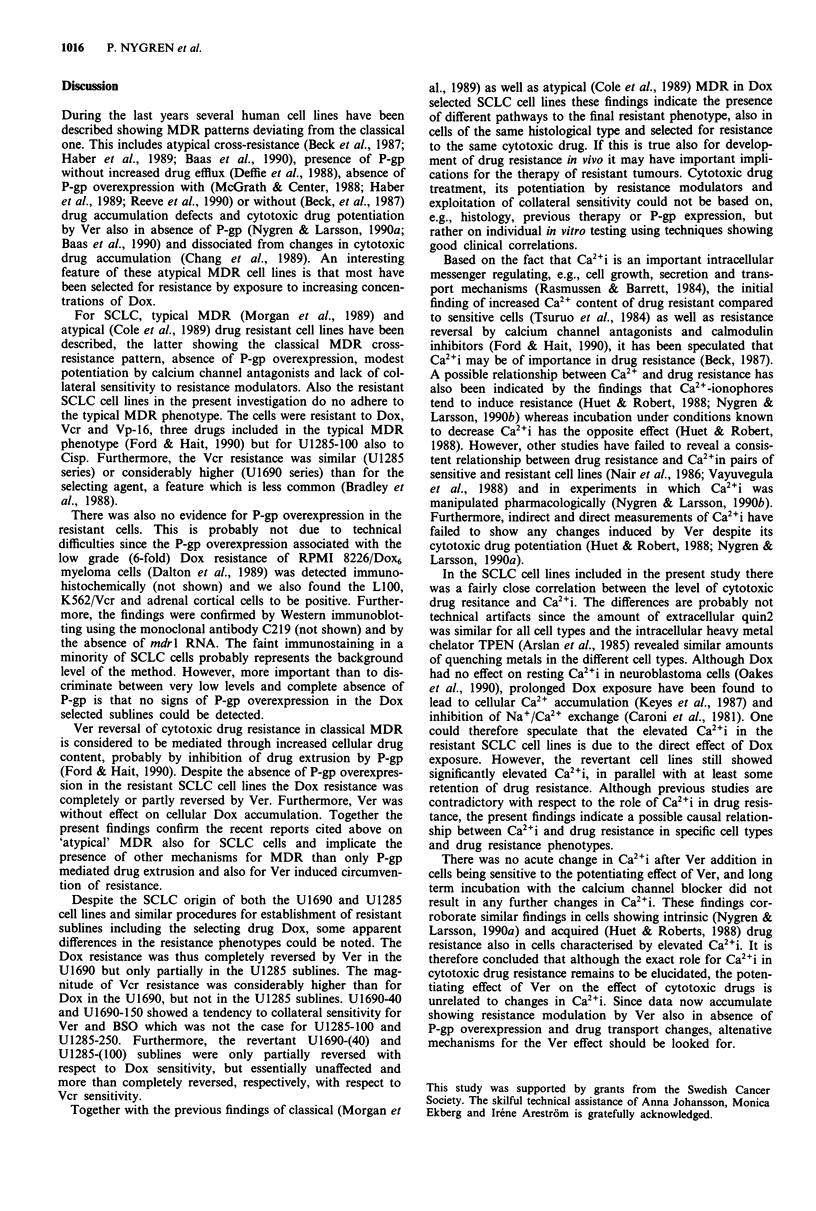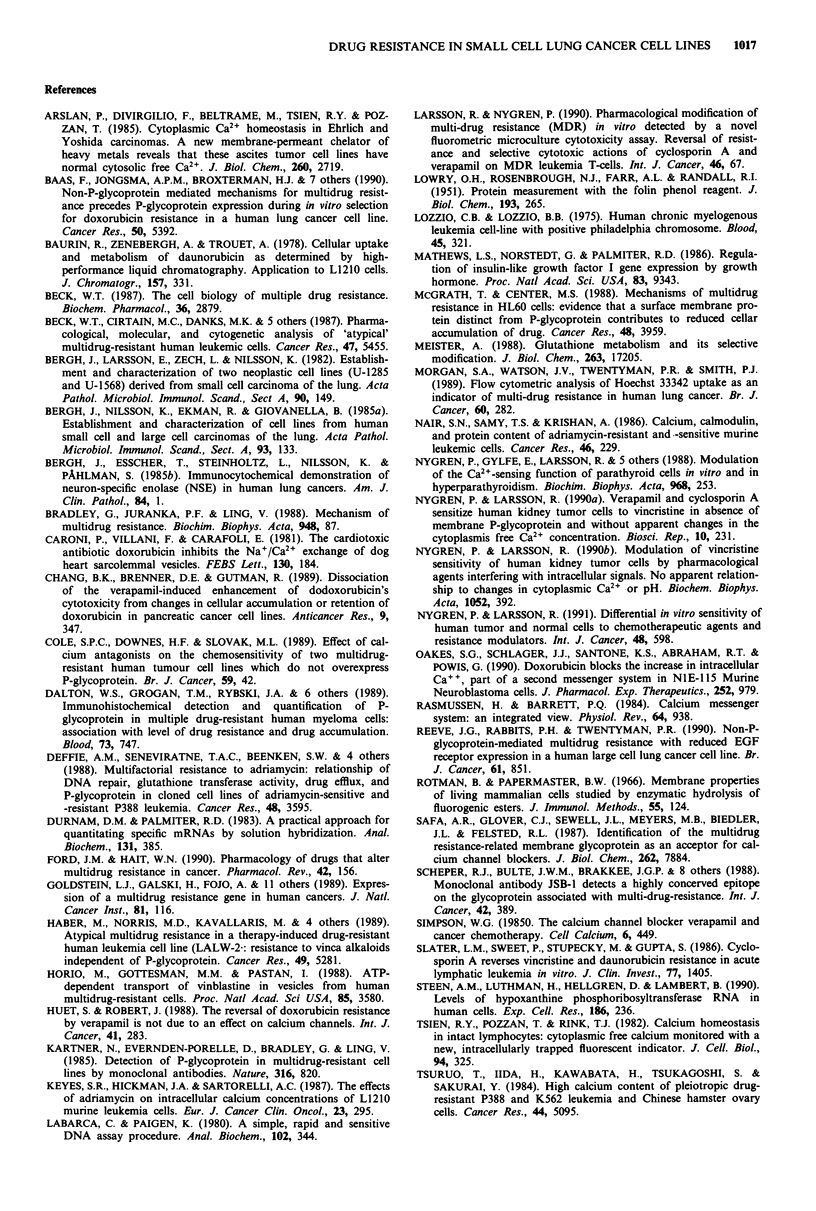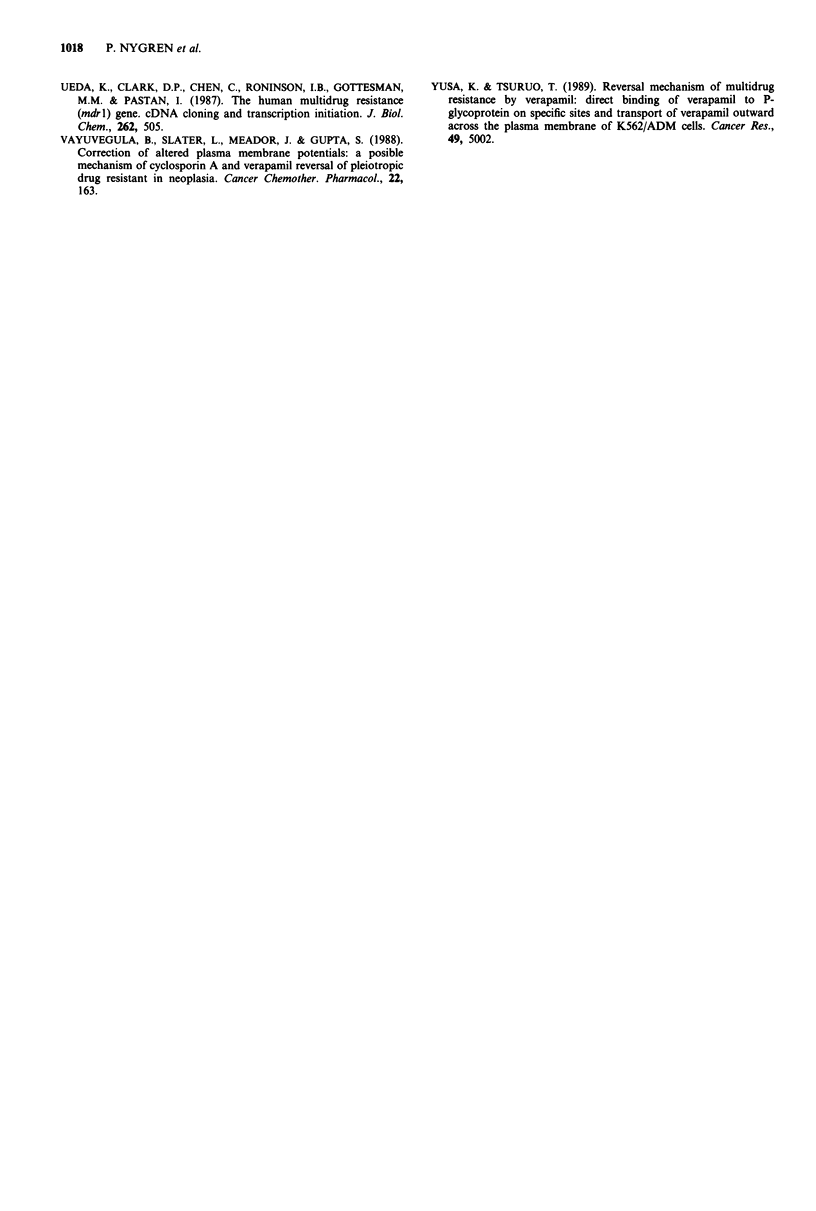# Doxorubicin selected multidrug-resistant small cell lung cancer cell lines characterised by elevated cytoplasmic Ca2+ and resistance modulation by verapamil in absence of P-glycoprotein overexpression.

**DOI:** 10.1038/bjc.1991.456

**Published:** 1991-12

**Authors:** P. Nygren, R. Larsson, A. Gruber, C. Peterson, J. Bergh

**Affiliations:** Department of Oncology, University Hospital, University of Uppsala, Sweden.

## Abstract

Sublines from the small cell lung cancer (SCLC) cell lines U1285 and U1690, denoted U1285-100, U1285-250, U1690-40 and U1690-150, were adapted to grow in the continuous presence of 100, 250, 40 and 150 ng ml-1 doxorubicin (Dox), respectively. The Dox resistance was accompanied by cross-resistance to vincristine (Vcr), Vp-16 and for U1285-100 also to cisplatinum. Sublines of U1690-40 and U1285-100, cultured in absence of Dox for 4 months were only partially reversed with respect to Dox resistance. Neither the parental nor the most Dox resistance sublines had detectable levels of mdr 1 RNA but a small fraction of cells in all cell lines stained weakly positive for P-glycoprotein (P-gp). Verapamil (Ver) at 5 microM reversed the Dox resistance completely and partly in the U1690 and U1285 sublines, respectively, but did not increase the cellular accumulation of Dox. The cytoplasmic free Ca2+ concentration (Ca2+i) was close to 100 nM in both parental cell lines but elevated in the U1285-100 and U1690-40 sublines by 21 and 44%, respectively, and in U1285-250 and U1690-150 by 51 and 91%, respectively. The partly reverted sublines still showed significant but smaller elevations in Ca2+i of 10-30%. Ver was without acute or long term effects of Ca2+i in the U1285-100 and U1690-40 sublines. Selection for Dox resistance in SCLC may thus result in atypical multidrug-resistance characterised by absence of P-gp overexpression and atypical cross-resistance. Although Ver did not seem to affect Dox accumulation it may still work as a resistance modulator.(ABSTRACT TRUNCATED AT 250 WORDS)


Br. J. Cancer (1991), 64, 1011-1018 © Macmillan Press Ltd., 1991

### Doxorubicin selected multidrug-resistant small cell lung cancer cell lines characterised by elevated cytoplasmic $Ca^{2+}$ and resistance modulation by verapamil in absence of P-glycoprotein overexpression

P. Nygren[1], R. Larsson[2], A. Gruber[3], C. Peterson[4] & J. Bergh[1]

*Departments of [1]Oncology and [2]Clinical Pharmacology, University Hospital, University of Uppsala, S-751 85 Uppsala, Sweden and Departments of [3]Medicine and [4]Clinical Pharmacology, Karolinska Hospital, S-104 01 Stockholm, Sweden.*

**Summary** Sublines from the small cell lung cancer (SCLC) cell lines U1285 and U1690, denoted U1285-100, U1285-250, U1690-40 and U1690-150, were adapted to grow in the continuous presence of 100, 250, 40 and 150 ng ml$^{-1}$ doxorubicin (Dox), respectively. The Dox resistance was accompanied by cross-resistance to vincristine (Vcr), Vp-16 and for U1285-100 also to cisplatinum. Sublines of U1690-40 and U1285-100, cultured in absence of Dox for 4 months were only partially reversed with respect to Dox resistance. Neither the parental nor the most Dox resistance sublines had detectable levels of *mdr*1 RNA but a small fraction of cells in all cell lines stained weakly positive for P-glycoprotein (P-gp). Verapamil (Ver) at 5 $\mu$M reversed the Dox resistance completely and partly in the U1690 and U1285 sublines, respectively, but did not increase the cellular accumulation of Dox. The cytoplasmic free $Ca^{2+}$ concentration ($Ca^{2+}$i) was close to 100 nM in both parental cell lines but elevated in the U1285-100 and U1690-40 sublines by 21 and 44%, respectively, and in U1285-250 and U1690-150 by 51 and 91%, respectively. The partly reverted sublines still showed significant but smaller elevations in $Ca^{2+}$i of 10–30%. Ver was without acute or long term effects of $Ca^{2+}$i in the U1285-100 and U1690-40 sublines. Selection for Dox resistance in SCLC may thus result in atypical multidrug-resistance characterised by absence of P-gp overexpression and atypical cross-resistance. Although Ver did not seem to affect Dox accumulation it may still work as a resistance modulator. There may be a role for increased $Ca^{2+}$i in drug resistance in SCLC cells, but resistance reversal by Ver seems unrelated also to changes in $Ca^{2+}$i.

Acquired cytotoxic drug resistance is often extended also to drugs not included in the treatment regimen and the most consistent finding *in vitro* in such multidrug-resistance (MDR) is resistance to anthracyclines, vinca alkaloids and epipodophyllotoxins with a decreased drug accumulation compared to the sensistive cells (Beck, 1987; Bradley *et al.*, 1988). *In vivo* (Goldstein *et al.*, 1989) as well as *in vitro* (Beck, 1987; Bradley *et al.*, 1988) the MDR phenotype is often characterised by expression of the 170 kDa membrane P-glycoprotein (P-gp) encoded by the *mdr*1 gene (Ueda *et al.*, 1987). Several lines of evidence indicate that this protein mediates an energy dependent extrusion of various cytotoxic drugs (Beck, 1987; Bradley *et al.*, 1988; Horio *et al.*, 1988).

Several well known pharmacological agents have been shown to reverse MDR *in vitro* (Ford & Hait, 1990). One of the most studied of these resistance modulators is the calcium channel blocker verapamil (Ver), which modulates acquired as well as intrinsic drug resistance of various cell types (Simpson, 1985). The mechanism for resistance reversal by Ver is still not completely understood although it has been found that Ver may compete with cytotoxic drugs for binding to the P-gp, resulting in a decrease in drug efflux and thus enhanced cytotoxicity (Safa *et al.*, 1987; Yusa & Tsuruo, 1989).

Transmembrane transport as well as many other cell functions are known to be regulated by intracellular signals such as, e.g. changes in the cytoplasmic free $Ca^{2+}$ concentration ($Ca^{2+}$i; Rasmussen & Barrett, 1984). Based on findings of increased calcium content of MDR cells (Tsuruo *et al.*, 1984) and resistance reversal by the calcium channel blocker Ver, it has been speculated that $Ca^{2+}$i may have role in cytotoxic drug resistance and that the sensitising effect of Ver could be due to a decrease in $Ca^{2+}$i (Beck, 1987).

In the present study some of these hypotheses were evaluted in two established human small cell lung cancer (SCLC) cell lines. We investigated whether development of resistance to doxorubicin (Dox) also conferred resistance to other cytotoxic drugs and was accompanied by increased expression of P-gp and changes in drug accumulation. The possible role of $Ca^{2+}$i in drug resistance was evaluated by quin2 measurements in sublines showing varying degrees of drug resistance. Furthermore, the possible potentiating effect of Ver on Dox cytotoxicity was evaluated and correlated to acute and long term changes in $Ca^{2+}$i.

### Materials and methods

### *Cell lines and culture*

The human U1285 and U1690 SCLC were established as described previously (Bergh *et al.*, 1982; Bergh *et al.*, 1985*a*). Both lines have a doubling time of about 3 days. By gradually increasing the Dox (Farmitalia Carlo Erba, Italy) concentration in the culture medium, sublines of U-1285 and U-1690, denoted U1285-100, U1285-250, U1690-40 and U1690-150 were adpated to grow with similar doubling times as the parental lines in the continuous presence of 100, 250, 40 and 150 ng ml$^{-1}$ Dox, respectively. Sublines from U1285-100 and U1690-40, denoted U1285-(100) and U1690-(40), respectively, were cultured in absence of Dox for 4 months prior to inclusion in the experiments and will be referred to as revertant cell lines. All cell lines were grown in RPMI 1640 medium (Flow Laboratories, Herts, England) containing 10% foetal calf serum (FSC; Flow) and antibiotics and were refed twice weakly. The cultures were incubated at 37°C in an atmosphere containing 5% $CO_2$ and 95% air. In control experiments for expression of P-gp, *mdr*1 RNA and drug accumulation the parental T-ALL cell line L0 and its vincristine (Vcr) resistant subline L100, kindly provided by Dr L. Slater, Department of Medicine, University of California, Irvine, CA (Slater *et al.*, 1986) as well as the chronic myelocytic leukaemia cell line K562 (Lozzio & Lozzio, 1975) and its subline K562/Vcr, adapted to growth in presence of 30 mM Vcr, were used. The L100 subline is

Correspondence: P. Nygren, Department of Oncology, University Hospital, University of Uppsala, S-751 85 Uppsala, Sweden.
Received 20 February 1990; and in revised form 14 August 1991.

approximately 160-fold Vcr resistant, shows cross-resistance to Dox and Vp-16, expresses P-gp and is modulated by Ver (Nygren & Larsson, 1991). The K562/Vcr subline is approximately 250-fold Vcr resistant and shares the other characteristics with the L100 subline (Nygren & Larsson, unpublished data).

For investigation of the cytotoxic drug sensitivity, 25,000 cells in 190 µl culture medium were seeded into each well of flat bottomed 96 well microtiter culture plates (Nunc, Roskilde, Denmark). The indicated concentrations of Dox, Vcr (Sigma Chemical Co., St. Louis, MO), cisplatinum (Cisp; Sigma) or Vp-16 (Bristol-Myers, Solna, Sweden), all dissolved in phosphate buffered saline (PBS), were then added to triplicate wells. For evaluation of a possible sensitising effect of Ver on Dox sensitivity and of the glutathione depletor buthionine sulfoximine (BSO; Sigma; Meister, 1988) on Cisp sensitivity, 5 µM Ver (in dimethyl sulfoxide/PBS; Sigma) or 1–10 µM BSO (in PBS) was added just prior to the cytotoxic drug. The volume of each added drug was always 10 µl giving a final maximal dimethyl sulfoxide concentration of 0.1%, which did not affect cell growth. The cells were then cultured for 72 h under the conditions described above. No medium change was done during the culture period.

*Measurement of cell survival*

Cell survival after culture was estimated by using the fluorogenic substrate fluorescein diacetate (FDA; Sigma) which rapidly enters intact cells where it is hydrolysed to its fluorescent derivative fluorescein (Rotman & Papermaster, 1966). The details of this technique have recently been described (Larsson & Nygren, 1990). Briefly, after culture the plates were centrifuged (100 *g*, 5 min), the medium removed by flicking the plate, and the wells washed once with 200 µl of the buffer described below. To each well was then added 200 µl of assay buffer containing 0.5 mM $Mg^{2+}$ , 1,25 mM $Ca^{2+}$, 3 mM glucose, 10 mM Hepes (pH 7.4) and physiologically balanced in other cations and with $Cl^-$ as the sole anion and with 10 µg $ml^{-1}$ FDA. The plates were then incubated for 60 min at 37°C after which the fluorescence from each well was read in a Fluoroscan II microfluorometer (Flow) with filters set at 485 and 538 nm for excitation and emission, respectively. The fluorometer was blanked against wells containing assay medium with dye. Each plate was read in about 1 min and the fluorescence data was then imported to a Macintosh SE computer for statistical and graphical processing.

Cell survival after culture is expressed as survival index (SI) defined as FDA fluorescence for treated wells/FDA fluorescence for untreated control wells expressed as per cent. $IC_{50}$ was defined as the cytotoxic drug concentration resulting in a SI of 50% of control. Resistance factor (RF) was defined as $IC_{50}$ for the subline/$IC_{50}$ for the parental line.

*Immunohistochemical staining for P-glycoprotein*

P-gp staining was performed using the monoclonal anti-P-gp antibody JSB-1 (Sanbio, Uden, The Netherlands; Scheper et al., 1988) with the technique described previously (Bergh et al., 1985*b*). Frozen sections (4–6 µM) of human adrenal cortex and cytocentrifuge preparations of MDR L100 and K562/Vcr cells were used as positive controls whereas sensitive L0 and K562 cells and preparations stained with the above technique, but without primary antibody served as negative controls. The specimens were incubated with the JSB-1 antibody diluted 1/30 in PBS for 60 min, followed by washing and application of a biotinylated rabbit-anti-mouse complex (Vector Laboratories Inc., Burlingame, CA) and then a avidin-biotinylated horseradish peroxidase complex (Vector Laboratories). The coverslips were then developed in 0.02% 3-amino-9 ethycarbazole supplemented with 0.002% $H_2O_2$.

The specimens were counterstained with Mayer's hematoxylin, mounted and judged –, +, ++ or +++ by light microscopy. In separate control experiments the monoclonal P-gp antibody C219 (Centocor, Malvern, PA; Kartner et al., 1985) was used instead of JSB-1.

*Measurement of mdr1 RNA*

Measurement of *mdr*1 mRNA was performed by hybridisation in solution as described (Durnam et al., 1983; Mathews et al., 1986; Steen et al., 1990) with K562 and K562/Vcr as controls. Nucleic acids extracts were prepared (Durnam et al., 1983) and aliquots were taken for determination of DNA content by Hoechst fluorometry (Labarca et al., 1980). Plasmid pGem-4 (Promega Corporation, Madison, WI) carrying 1383 basepairs of the *mdr*1 cDNA sequence (pHDR5A) was kindly provided by Drs M. Gottesman and I. Pastan, NCI, Bethesda, MD (Ueda et al., 1987). A 393 basepair long sequence was subcloned into a new plasmid (pGem-3Zf (+); Promega). A 403 nucleotides long antisense probe was generated by transcription of Stu I (New England Biolabs, Beverly, USA) cleaved pHDR5A with SP6 RNA polymerase (Promega) in the presence of [S35]UTP (>37 × $10^6$ MBq $mmol^{-1}$; Amersham International, Amersham, England). A 439 nucleotides long unlabelled sense RNA (complementary to the labelled antisense probe) was transcribed by Sp6 RNA polymerase from *Eco*RI (New England Biolabs) cleaved pGem-3Zf (+). The concentration of the unlabelled RNA was determined spectrophotometrically at 260 nm.

Aliquots of the extracts or unlabelled sense RNA were adjusted to 20 µl with 0.2 × SET (1 × SET is 1% sodium dodecyl sulfate, 10 mM ethylenediaminetetraacetic acid (EDTA) and 20 mM Tris-HCl, pH 7.5) and mixed with 30,000 counts per min (c.p.m.) of antisense probe dissolved in 20 µl hybridisation solution (0.6 M NaCl, 4 mM EDTA, 7.5 mM dithiothreitol, 25% deionised recrystallised formamide and 20 mM Tris-HCl, pH 7.5) and incubated for 18 h at 68°C. Subsequently, the samples were treated with 1 ml of an RNase solution of 40 µg $ml^{-1}$ RNase A (Sigma), 2 µg $ml^{-1}$ RNase T1 (Sigma), 100 µg $ml^{-1}$ salmon sperm DNA (Sigma), 0.3 M NaCl, 2 mM EDTA, 10 mM Tris–HCl (pH 7.5) and incubated for 45 min at 37°C. After addition of 100 µl 100% trichloroacetic acid, samples were kept on ice for 30 min and the RNase resistant precipitates were collected on Whatman GF/C filters (Whatman International, Maidstone, England). After addition of 4 ml Insta-gel scintillation liquid (Packard Instrument Company, Downers Grove, ILL) the radioactivity was determined in a liquid scintillation counter (Packard).

The quantities of *mdr*1 RNa in the extracts were determined by comparison with a standard curve, generated by hybridisation with increasing amounts of the unlabelled sense (Steen et al., 1990). Samples classified as positive for *mdr*1 RNA show at least twice the background radioactivity and a proportional increase in radioactivity with increasing amounts of added extracts. The results are presented as c.p.m. with background subtracted for three dilutions of each extract. Based on the standard curve, a molecular weight of sense RNA of 1.49 × $10^5$, c.p.m. $\mu g^{-1}$ DNA values for each extract and the assumption of a DNA content of 6 pg $cell^{-1}$, the number of *mdr*1 RNa copies/cell may be calculated. The detection limit in a sample containing 30 µg DNA is 7.5 × $10^5$ copies of RNA which corresponds to 0.15 RNA copies $cell^{-1}$.

*Measurement of cellular doxorubicin accumulation*

The parental and most resistant sublines were compared with respect to Dox accumulation in absence and presence of Ver. The cells were incubated at 37°C for 1 h at a density of 1.5 × $10^6$ cells $ml^{-1}$ in RPMI 1640 medium supplemented with 10% newborn calf serum (Gibco, Paisley, Scotland) and containing 1 µM Dox and with or without 5 µM Ver. The incubation was stopped by mixing 1.5 ml of the incubate with 5 ml ice cold PBS. After two washes in ice cold PBS the cell pellets were kept in −20°C until analysis.

Cellular concentrations of Dox were determined by high-performance liquid chromatography as described (Baurin et

*al.*, 1978). Briefly, after sonication of each cell pellet in 0.5 ml PBS a 0.2 ml aliquit was added to 0.2 ml 0.1 M borate buffer (pH 9.8) containing 1 μM daunorubicin as internal standard. Extraction was made by addition of 1.8 ml chloroform/methanol (4:1 by volume) after which the drugs were separated on a Lichosorb Si-60 column (Hibar; Merck, Darmstadt, Germany) and eluted with a mixture of chloroform, methanol, glacial acetic acid and 0.3 mM $MgCl_2$ (720:210:40:30 by volume) at a flow rate of 1.5 ml $min^{-1}$. The drugs were then quantified by fluorometry at 480 and 560 nm for excitation and emission wavelengths, respectively.

The protein content in the dissolved cell pellets was determined by the method described by Lowry (Lowry *et al.*, 1951) and the cellular accumulation of Dox is expressed as nmol Dox $mg^{-1}$ protein.

### *Measurement of the cytoplasmic free $Ca^{2+}$ concentration*

Fluorometric measurements of $Ca^{2+}$i were performed as described previously (Nygren *et al.*, 1988). Briefly, $5 \times 10^6$ quin2 (Sigma; Tsien *et al.*, 1982) loaded cells were suspended in 1.3 ml of the buffer used for cell survival measurements and incubated with constant stirring at 37°C in a 1 cm cuvette of a Perkin-Elmer LS5 spectrofluorometer with excitation and emission wavelengths set at 339 and 492 nm, respectively. Ver was added from a 100-fold concentrated stock solution in DMSO/buffer. In the measurement of the long term effect of Ver on $Ca^{2+}$i, 5 μM Ver was present also in the physiological buffer used for washing and assay. The addition of vehicle was without effect on quin2 fluorescence. Calibration for calculation of $Ca^{2+}$i was then performed as described (Nygren *et al.*, 1988). Extracellular quin2 never exceeded 7% of $F_{max}$ as judged by the fall of fluorescence signal after addition of 3 mM EGTA and was similar in all cell lines. The increase in fluorescence upon addition of the intracellular heavy metal chelator TPEN (Calbiochem, La Jolla, CA; Arslan *et al.*, 1985) corresponds to a rise in $Ca^{2+}$i of 30–40 nM in all cell lines, indicating no significant differences in quenching heavy metal content.

Student's paired *t*-test was used for statistical comparisons.

### Results

### *Cytotoxic drug sensitivity and effects of resistance modulators*

The $IC_{50}$ values for Dox were 0.4 and 0.2 μg $ml^{-1}$ for the parental U1690 and U1285 cell lines, respectively (Table I). The resistant sublines U1690-40 and U1690-150 were approximately 6 and 7-fold resistant to Dox whereas the revertant subline U1690-(40) retained most of its Dox resistance with a RF of 4. The corresponding values for U1285-100 and U1285-250 were 18 and 16, respectively, whereas the revertant U1285-(100) showed a RF of 8. The sublines also developed cross-resistance to Vcr, which for the U1690 sublines was of even higher magnitude than for Dox with RFs of 40 and 246 for U1690-40 and U1690-150, respectively. Again the U1690-(40) revertant subline retained much of its original resistance with a RF of 34. The corresponding values for U1285-100 and U1285-250 were 5 and 17, respectively. The revertant U1285-(100) cells showed a paradoxical 2-fold increase in Vcr sensitivity compared to the parental cell line. The U1690-40 and U1285-100 sublines were also cross-resistant to Vp-16 with RFs of 16 and 8, respectively. To check for cross-resistant to a cytotoxic agent not included in the typical MDR phenotype we also investigated two sublines for Cisp sensitivity. U1690-40 and U1285-100 were found 1.4 and 4-fold resistant compared to the parental lines.

Ver at 5 μM reversed the Dox resistance completely in the U1690 sublines and partly in the U1285 sublines (Table I). BSO at 1–1 μM, depending on the sensitivity of the cell lines to BSO alone, increased the Cisp sensitivity 1.3–5-fold in parental and resistant cells.

The U1690 sublines showed minor collateral sensitivity to both Ver and BSO with normalisation to the parental

**Table I** $IC_{50}$:s for inhibition of SI by Dox, Vcr, Vp-16 and Cisp in parental and resistant U1690 and U1285 cell lines[a]

| | Cell line | | | | | | | |
|---|---|---|---|---|---|---|---|---|
| *Cytotoxic agent* | *U1690* | *U1690-40* | *U1690-150* | *U1690-(40)* | *U1285* | *U1285-100* | *U1285-250* | *U1285-(100)* |
| Dox (μg $ml^{-1}$) | 0.4 ± 0.1 | 2.2 ± 0.4 (5.5) | 2.8 ± 0.7 (7.0) | 1.4 ± 0.3 (3.5) | 0.2 ± 0.1 | 3.4 ± 0.4 (17.6) | 3.1 ± 0.7 (15.5) | 1.6 ± 0.2 (8.0) |
| Dox + 5 μM Ver | 0.3 ± 0.1 | 0.3 ± 0.1 | 0.3 ± 0.0 | 0.3 ± 0.0 | 0.2 ± 0.0 | 1.1 ± 0.3 | 0.7 ± 0.3 | 1.0 ± 0.3 |
| Vcr (ng $ml^{-1}$) | 0.9 ± 0.3 | 36.1 ± 11.6 (40.1) | 221 ± 74 (245.6) | 30.6 ± 14.2 (34.0) | 9.1 ± 5.5 | 44.6 ± 20.9 (4.9) | 152 ± 74 (16.7) | 4.4 ± 1.4 (0.5) |
| Vp-16 (μM) | 4.1 ± 0.6 | 67.0 ± 7.1 (16.3) | ND | ND | 5.5 ± 0.6 | 45.1 ± 5.6 (8.2) | ND | ND |
| Cisp (ng $ml^{-1}$) | 23.2 ± 4.1 | 32.5 ± 3.5 (1.4) | ND | ND | 10.6 ± 6.6 | 38.5 ± 7.6 (3.6) | ND | ND |
| Cisp + BSO[b] | 5.9 ± 2.1 | 10.2 ± 2.6 | ND | ND | 8.1 ± 4.8 | 7.7 ± 3.5 | ND | ND |

[a]Mean values ± SE of triplicate wells of 4–10 independent experiments. [b]The concentration of BSO used was: 1 μM (U1285), 5 μM (U1690) or 10 μM (U1285-100 and U1690-40). Resistance factors for Dox, Vcr and Cisp are shown within parentheses. ND, not done.

phenotype in the revertant subline (Figure 1). In the U1285 series of cell lines there was no evidence for collateral sensitivity or cross-resistance to these modulators (Figure 2).

*Expression of P-glycoprotein*

As expected adrenal cortex stained strongly positive for P-gp (Table II). The parental L0 and K562 cells were found negative whereas the majority of the MDR L100 and K562/Vcr cells stained positive, although weaker than adrenal cortical cells. Both the U1690 and U1285 cell lines as well as their drug resistant sublines showed faint membrane staining in a small proportion (<15%) of the cells. Qualitatively similar results were obtained in control experiments using the monoclonal antibody C219 (not shown). Furthermore, Western blotting confirmed expression of a protein reactive with the C219 antibody in L100 cells whereas no such protein was found in the L0 cells or any of the SCLC cell lines (not shown).

*Expression of* mdr*1 RNA*

Data for hybridisation between labelled RNA probe and unlabelled sense RNA (standard curve) or nucleic acid extract from K562/Vcr are shown in Figure 3a. There is a proportional increase in radioactivity with increasing amounts of added sense RNA or extract. The c.p.m. for K562/Vcr corresponds to approximately 98 *mdr*1 RNA copies/cell. For parental K562 cells as well as the parental and most resistant SCLC sublines the c.p.m. values were just above background and the *mdr*1 RNA content of these samples was below the detection limit of the assay (Figure 3b).

*Cellular doxorubicin accumulation*

The U1690-150 and U1285-250 sublines accumulated somewhat more and less Dox, respectively, compared to their parental cell lines (Figure 4). Presence of 5 μM Ver during incubation did not affect the Dox accumulation in any cell line. In control experiments using the same method, K562/Vcr cells were found to accumulate 50% less daunorubicin compared to the parental cells and presence of Ver during incubation normalised this accumulation defect (not shown).

*Cytoplasmic free $Ca^{2+}$ concentrations*

Basal $Ca^{2+}$i of U1690 cells was 100 nM and was increased to 144 and 191 nM for the U1690-40 and U1690-150 sublines, respectively (Figure 5a; $P < 0{,}001$ *vs* parental cells). The revertant subline U1690-(40) showed a $Ca^{2+}$i 30 nM higher than the U1690 cells ($P < 0{,}02$). The $Ca^{2+}$i differences were

**Figure 1** Effects of increasing concentrations of Ver **a**, and BSO, **b**, in the parental U1690 cell line and its resistant sublines. The indicated concentrations of Ver or BSO were added in triplicate wells at day 0 and the cultures then incubated for 72 h followed by determination of SI as described in Materials and methods. Mean values ± s.e. of 4–7 experiments. □ U1690; ■ U1690-40; ● U1690-150; ▲ U1690-(40).

qualitatively similar in the U1285 series of cell lines, although quantitatively less with $Ca^{2+}$i values of 106, 128 ($P < 0{,}01$), 160 ($P < 0{,}001$) and 117 nM ($P < 0{,}05$) for the U1285, U1285-100, U1285-250 and U1285-(100) cell lines, respectively (Figure 5b).

Addition of 5 μM Ver was without acute (within 3 min) effect on $Ca^{2+}$i in the parental cell lines and the U1690-40 and U1285-100 sublines (Figure 6). After incubation with 5 μM Ver for 48 h, $Ca^{2+}$i was slightly decreased by 13 nM in U1285 cells whereas $Ca^{2+}$i in the other cell lines was not significantly altered.

**Table II** Expression of immunohistochemically detectable P-glycoprotein[a]

| *Tissue/Cell type* | *Staining reaction* | *% of cells positive* | *Region positive* |
|---|---|---|---|
| Adrenal cortex | + + + | 75–100 | Membrane |
| L0 | – | – | – |
| L100 | + + | 75–100 | Membrane/Golgi |
| K562 | – | – | – |
| K562/Vcr | + + | 75–100 | Membrane |
| U1285 | + | <15 | Membrane |
| U1285-100 | + | <15 | Membrane |
| U1285-250 | + | <15 | Membrane |
| U1285-(100) | + | <15 | Membrane |
| U1690 | + | <15 | Membrane |
| U1690-40 | + | <15 | Membrane |
| U1690-150 | + | <15 | Membrane |
| U1690-(40) | + | <15 | Membrane |

[a]Immunohistochemical stainings were performed as described in Materials and methods using the monoclonal antibody JSB-1. Intensity of staining is in comparison to adrenal cortex which was judged as + + +. The fraction of cells positive was determined by examination of 100 cells in each experiment. Data from one typical experiment of 4.

**Figure 2** Effects of increasing concentrations of Ver **a**, and BSO **b**, in the parental U1285 cell line and its resistant sublines. The indicated concentrations or Ver of BSO were added in triplicate wells at day 0 and the cultures then incubated for 72 h followed by determination of SI as described in Materials and methods. Mean values ± s.e. of 4–7 experiments. □ U1285; ■ U1285-100; ● U1285-250; ▲ U1285-(100).

**Figure 3** Hybridisation between labelled *mdr*1 RNA probe and increasing amounts of unlabelled sense RNA (standard curve, inset) or nucleic acid extract from K562/Vcr cells **a**. The corresponding data for the parental K562 cells as well as for the parental and most resistant SCLC cell lines indicate *mdr*1 RNA levels below the detection limit of the assay **b**. Data from one typical experiment. ○ K562; □ U1690; ■ U1690-150; △ U1285; ▲ U1285-250.

**Figure 4** Dox accumulation of U1690 and U1285 cell lines and their most resistant sublines after incubation in 1 μM Dox for 1 h in absence (open bars) or presence (hatched bars) of 5 μM Ver. Mean ± s.e. for six samples.

**Figure 5** Steady state $Ca^{2+}i$ of U1690 and U1285 cell lines and their resistant sublines. $Ca^{2+}i$ was measured fluorometrically using quin2 as described in Materials and methods. Mean ± s.e. for 7–17 experiments, each performed in duplicates.

**Figure 6** $Ca^{2+}i$ steady-state deviation from $Ca^{2+}i$ of unexposed control cells in U1285, U1285-100, U1690 and U1690-40 cells immediately after addition of 5 μM Ver (open bars) and after exposure of the cells to 5 μM Ver for 48 h during culture (hatched bars). Mean ± s.e. of 3–6 experiments. *$P$ <0.001.

### Discussion

During the last years several human cell lines have been described showing MDR patterns deviating from the classical one. This includes atypical cross-resistance (Beck et al., 1987; Haber et al., 1989; Baas et al., 1990), presence of P-gp without increased drug efflux (Deffie et al., 1988), absence of P-gp overexpression with (McGrath & Center, 1988; Haber et al., 1989; Reeve et al., 1990) or without (Beck, et al., 1987) drug accumulation defects and cytotoxic drug potentiation by Ver also in absence of P-gp (Nygren & Larsson, 1990a; Baas et al., 1990) and dissociated from changes in cytotoxic drug accumulation (Chang et al., 1989). An interesting feature of these atypical MDR cell lines is that most have been selected for resistance by exposure to increasing concentrations of Dox.

For SCLC, typical MDR (Morgan et al., 1989) and atypical (Cole et al., 1989) drug resistant cell lines have been described, the latter showing the classical MDR cross-resistance pattern, absence of P-gp overexpression, modest potentiation by calcium channel antagonists and lack of collateral sensitivity to resistance modulators. Also the resistant SCLC cell lines in the present investigation do no adhere to the typical MDR phenotype. The cells were resistant to Dox, Vcr and Vp-16, three drugs included in the typical MDR phenotype (Ford & Hait, 1990) but for U1285-100 also to Cisp. Furthermore, the Vcr resistance was similar (U1285 series) or considerably higher (U1690 series) than for the selecting agent, a feature which is less common (Bradley et al., 1988).

There was also no evidence for P-gp overexpression in the resistant cells. This is probably not due to technical difficulties since the P-gp overexpression associated with the low grade (6-fold) Dox resistance of RPMI 8226/$Dox_6$ myeloma cells (Dalton et al., 1989) was detected immunohistochemically (not shown) and we also found the L100, K562/Vcr and adrenal cortical cells to be positive. Furthermore, the findings were confirmed by Western immunoblotting using the monoclonal antibody C219 (not shown) and by the absence of *mdr*1 RNA. The faint immunostaining in a minority of SCLC cells probably represents the background level of the method. However, more important than to discriminate between very low levels and complete absence of P-gp is that no signs of P-gp overexpression in the Dox selected sublines could be detected.

Ver reversal of cytotoxic drug resistance in classical MDR is considered to be mediated through increased cellular drug content, probably by inhibition of drug extrusion by P-gp (Ford & Hait, 1990). Despite the absence of P-gp overexpression in the resistant SCLC cell lines the Dox resistance was completely or partly reversed by Ver. Furthermore, Ver was without effect on cellular Dox accumulation. Together the present findings confirm the recent reports cited above on 'atypical' MDR also for SCLC cells and implicate the presence of other mechanisms for MDR than only P-gp mediated drug extrusion and also for Ver induced circumvention of resistance.

Despite the SCLC origin of both the U1690 and U1285 cell lines and similar procedures for establishment of resistant sublines including the selecting drug Dox, some apparent differences in the resistance phenotypes could be noted. The Dox resistance was thus completely reversed by Ver in the U1690 but only partially in the U1285 sublines. The magnitude of Vcr resistance was considerably higher than for Dox in the U1690, but not in the U1285 sublines. U1690-40 and U1690-150 showed a tendency to collateral sensitivity for Ver and BSO which was not the case for U1285-100 and U1285-250. Furthermore, the revertant U1690-(40) and U1285-(100) sublines were only partially reversed with respect to Dox sensitivity, but essentially unaffected and more than completely reversed, respectively, with respect to Vcr sensitivity.

Together with the previous findings of classical (Morgan et al., 1989) as well as atypical (Cole et al., 1989) MDR in Dox selected SCLC cell lines these findings indicate the presence of different pathways to the final resistant phenotype, also in cells of the same histological type and selected for resistance to the same cytotoxic drug. If this is true also for development of drug resistance *in vivo* it may have important implications for the therapy of resistant tumours. Cytotoxic drug treatment, its potentiation by resistance modulators and exploitation of collateral sensitivity could not be based on, e.g., histology, previous therapy or P-gp expression, but rather on individual *in vitro* testing using techniques showing good clinical correlations.

Based on the fact that $Ca^{2+}i$ is an important intracellular messenger regulating, e.g., cell growth, secretion and transport mechanisms (Rasmussen & Barrett, 1984), the initial finding of increased $Ca^{2+}$ content of drug resistant compared to sensitive cells (Tsuruo et al., 1984) as well as resistance reversal by calcium channel antagonists and calmodulin inhibitors (Ford & Hait, 1990), it has been speculated that $Ca^{2+}i$ may be of importance in drug resistance (Beck, 1987). A possible relationship between $Ca^{2+}$ and drug resistance has also been indicated by the findings that $Ca^{2+}$-ionophores tend to induce resistance (Huet & Robert, 1988; Nygren & Larsson, 1990b) whereas incubation under conditions known to decrease $Ca^{2+}i$ has the opposite effect (Huet & Robert, 1988). However, other studies have failed to reveal a consistent relationship between drug resistance and $Ca^{2+}$in pairs of sensitive and resistant cell lines (Nair et al., 1986; Vayuvegula et al., 1988) and in experiments in which $Ca^{2+}i$ was manipulated pharmacologically (Nygren & Larsson, 1990b). Furthermore, indirect and direct measurements of $Ca^{2+}i$ have failed to show any changes induced by Ver despite its cytotoxic drug potentiation (Huet & Robert, 1988; Nygren & Larsson, 1990a).

In the SCLC cell lines included in the present study there was a fairly close correlation between the level of cytotoxic drug resitance and $Ca^{2+}i$. The differences are probably not technical artifacts since the amount of extracellular quin2 was similar for all cell types and the intracellular heavy metal chelator TPEN (Arslan et al., 1985) revealed similar amounts of quenching metals in the different cell types. Although Dox had no effect on resting $Ca^{2+}i$ in neuroblastoma cells (Oakes et al., 1990), prolonged Dox exposure have been found to lead to cellular $Ca^{2+}$ accumulation (Keyes et al., 1987) and inhibition of $Na^+/Ca^{2+}$ exchange (Caroni et al., 1981). One could therefore speculate that the elevated $Ca^{2+}i$ in the resistant SCLC cell lines is due to the direct effect of Dox exposure. However, the revertant cell lines still showed significantly elevated $Ca^{2+}i$, in parallel with at least some retention of drug resistance. Although previous studies are contradictory with respect to the role of $Ca^{2+}i$ in drug resistance, the present findings indicate a possible causal relationship between $Ca^{2+}i$ and drug resistance in specific cell types and drug resistance phenotypes.

There was no acute change in $Ca^{2+}i$ after Ver addition in cells being sensitive to the potentiating effect of Ver, and long term incubation with the calcium channel blocker did not result in any further changes in $Ca^{2+}i$. These findings corroborate similar findings in cells showing intrinsic (Nygren & Larsson, 1990a) and acquired (Huet & Roberts, 1988) drug resistance also in cells characterised by elevated $Ca^{2+}i$. It is therefore concluded that although the exact role for $Ca^{2+}i$ in cytotoxic drug resistance remains to be elucidated, the potentiating effect of Ver on the effect of cytotoxic drugs is unrelated to changes in $Ca^{2+}i$. Since data now accumulate showing resistance modulation by Ver also in absence of P-gp overexpression and drug transport changes, altenative mechanisms for the Ver effect should be looked for.

This study was supported by grants from the Swedish Cancer Society. The skilful technical assistance of Anna Johansson, Monica Ekberg and Iréne Areström is gratefully acknowledged.

### References

ARSLAN, P., DIVIRGILIO, F., BELTRAME, M., TSIEN, R.Y. & POZZAN, T. (1985). Cytoplasmic $Ca^{2+}$ homeostasis in Ehrlich and Yoshida carcinomas. A new membrane-permeant chelator of heavy metals reveals that these ascites tumor cell lines have normal cytosolic free $Ca^{2+}$. *J. Biol. Chem.*, **260**, 2719.

BAAS, F., JONGSMA, A.P.M., BROXTERMAN, H.J. & 7 others (1990). Non-P-glycoprotein mediated mechanisms for multidrug resistance precedes P-glycoprotein expression during *in vitro* selection for doxorubicin resistance in a human lung cancer cell line. *Cancer Res.*, **50**, 5392.

BAURIN, R., ZENEBERGH, A. & TROUET, A. (1978). Cellular uptake and metabolism of daunorubicin as determined by high-performance liquid chromatography. Application to L1210 cells. *J. Chromatogr.*, **157**, 331.

BECK, W.T. (1987). The cell biology of multiple drug resistance. *Biochem. Pharmacol.*, **36**, 2879.

BECK, W.T., CIRTAIN, M.C., DANKS, M.K. & 5 others (1987). Pharmacological, molecular, and cytogenetic analysis of 'atypical' multidrug-resistant human leukemic cells. *Cancer Res.*, **47**, 5455.

BERGH, J., LARSSON, E., ZECH, L. & NILSSON, K. (1982). Establishment and characterization of two neoplastic cell lines (U-1285 and U-1568) derived from small cell carcinoma of the lung. *Acta Pathol. Microbiol. Immunol. Scand., Sect A*, **90**, 149.

BERGH, J., NILSSON, K., EKMAN, R. & GIOVANELLA, B. (1985*a*). Establishment and characterization of cell lines from human small cell and large cell carcinomas of the lung. *Acta Pathol. Microbiol. Immunol. Scand., Sect. A*, **93**, 133.

BERGH, J., ESSCHER, T., STEINHOLTZ, L., NILSSON, K. & PÅHLMAN, S. (1985*b*). Immunocytochemical demonstration of neuron-specific enolase (NSE) in human lung cancers. *Am. J. Clin. Pathol.*, **84**, 1.

BRADLEY, G., JURANKA, P.F. & LING, V. (1988). Mechanism of multidrug resistance. *Biochim. Biophys. Acta*, **948**, 87.

CARONI, P., VILLANI, F. & CARAFOLI, E. (1981). The cardiotoxic antibiotic doxorubicin inhibits the $Na^+/Ca^{2+}$ exchange of dog heart sarcolemmal vesicles. *FEBS Lett.*, **130**, 184.

CHANG, B.K., BRENNER, D.E. & GUTMAN, R. (1989). Dissociation of the verapamil-induced enhancement of dodoxorubicin's cytotoxicity from changes in cellular accumulation or retention of doxorubicin in pancreatic cancer cell lines. *Anticancer Res.*, **9**, 347.

COLE, S.P.C., DOWNES, H.F. & SLOVAK, M.L. (1989). Effect of calcium antagonists on the chemosensitivity of two multidrug-resistant human tumour cell lines which do not overexpress P-glycoprotein. *Br. J. Cancer*, **59**, 42.

DALTON, W.S., GROGAN, T.M., RYBSKI, J.A. & 6 others (1989). Immunohistochemical detection and quantification of P-glycoprotein in multiple drug-resistant human myeloma cells: association with level of drug resistance and drug accumulation. *Blood*, **73**, 747.

DEFFIE, A.M., SENEVIRATNE, T.A.C., BEENKEN, S.W. & 4 others (1988). Multifactorial resistance to adriamycin: relationship of DNA repair, glutathione transferase activity, drug efflux, and P-glycoprotein in cloned cell lines of adriamycin-sensitive and -resistant P388 leukemia. *Cancer Res.*, **48**, 3595.

DURNAM, D.M. & PALMITER, R.D. (1983). A practical approach for quantitating specific mRNAs by solution hybridization. *Anal. Biochem.*, **131**, 385.

FORD, J.M. & HAIT, W.N. (1990). Pharmacology of drugs that alter multidrug resistance in cancer. *Pharmacol. Rev.*, **42**, 156.

GOLDSTEIN, L.J., GALSKI, H., FOJO, A. & 11 others (1989). Expression of a multidrug resistance gene in human cancers. *J. Natl. Cancer Inst.*, **81**, 116.

HABER, M., NORRIS, M.D., KAVALLARIS, M. & 4 others (1989). Atypical multidrug resistance in a therapy-induced drug-resistant human leukemia cell line (LALW-2·: resistance to vinca alkaloids independent of P-glycoprotein. *Cancer Res.*, **49**, 5281.

HORIO, M., GOTTESMAN, M.M. & PASTAN, I. (1988). ATP-dependent transport of vinblastine in vesicles from human multidrug-resistant cells. *Proc. Natl Acad. Sci USA*, **85**, 3580.

HUET, S. & ROBERT, J. (1988). The reversal of doxorubicin resistance by verapamil is not due to an effect on calcium channels. *Int. J. Cancer*, **41**, 283.

KARTNER, N., EVERNDEN-PORELLE, D., BRADLEY, G. & LING, V. (1985). Detection of P-glycoprotein in multidrug-resistant cell lines by monoclonal antibodies. *Nature*, **316**, 820.

KEYES, S.R., HICKMAN, J.A. & SARTORELLI, A.C. (1987). The effects of adriamycin on intracellular calcium concentrations of L1210 murine leukemia cells. *Eur. J. Cancer Clin. Oncol.*, **23**, 295.

LABARCA, C. & PAIGEN, K. (1980). A simple, rapid and sensitive DNA assay procedure. *Anal. Biochem.*, **102**, 344.

LARSSON, R. & NYGREN, P. (1990). Pharmacological modification of multi-drug resistance (MDR) *in vitro* detected by a novel fluorometric microculture cytotoxicity assay. Reversal of resistance and selective cytotoxic actions of cyclosporin A and verapamil on MDR leukemia T-cells. *Int. J. Cancer*, **46**, 67.

LOWRY, O.H., ROSENBROUGH, N.J., FARR, A.L. & RANDALL, R.I. (1951). Protein measurement with the folin phenol reagent. *J. Biol. Chem.*, **193**, 265.

LOZZIO, C.B. & LOZZIO, B.B. (1975). Human chronic myelogenous leukemia cell-line with positive philadelphia chromosome. *Blood*, **45**, 321.

MATHEWS, L.S., NORSTEDT, G. & PALMITER, R.D. (1986). Regulation of insulin-like growth factor I gene expression by growth hormone. *Proc. Natl Acad. Sci. USA*, **83**, 9343.

MCGRATH, T. & CENTER, M.S. (1988). Mechanisms of multidrug resistance in HL60 cells: evidence that a surface membrane protein distinct from P-glycoprotein contributes to reduced cellar accumulation of drug. *Cancer Res.*, **48**, 3959.

MEISTER, A. (1988). Glutathione metabolism and its selective modification. *J. Biol. Chem.*, **263**, 17205.

MORGAN, S.A., WATSON, J.V., TWENTYMAN, P.R. & SMITH, P.J. (1989). Flow cytometric analysis of Hoechst 33342 uptake as an indicator of multi-drug resistance in human lung cancer. *Br. J. Cancer*, **60**, 282.

NAIR, S.N., SAMY, T.S. & KRISHAN, A. (1986). Calcium, calmodulin, and protein content of adriamycin-resistant and -sensitive murine leukemic cells. *Cancer Res.*, **46**, 229.

NYGREN, P., GYLFE, E., LARSSON, R. & 5 others (1988). Modulation of the $Ca^{2+}$-sensing function of parathyroid cells *in vitro* and in hyperparathyroidism. *Biochim. Biophys. Acta*, **968**, 253.

NYGREN, P. & LARSSON, R. (1990*a*). Verapamil and cyclosporin A sensitize human kidney tumor cells to vincristine in absence of membrane P-glycoprotein and without apparent changes in the cytoplasmis free $Ca^{2+}$ concentration. *Biosci. Rep.*, **10**, 231.

NYGREN, P. & LARSSON, R. (1990*b*). Modulation of vincristine sensitivity of human kidney tumor cells by pharmacological agents interfering with intracellular signals. No apparent relationship to changes in cytoplasmic $Ca^{2+}$ or pH. *Biochem. Biophys. Acta*, **1052**, 392.

NYGREN, P. & LARSSON, R. (1991). Differential *in vitro* sensitivity of human tumor and normal cells to chemotherapeutic agents and resistance modulators. *Int. J. Cancer*, **48**, 598.

OAKES, S.G., SCHLAGER, J.J., SANTONE, K.S., ABRAHAM, R.T. & POWIS, G. (1990). Doxorubicin blocks the increase in intracellular $Ca^{++}$, part of a second messenger system in N1E-115 Murine Neuroblastoma cells. *J. Pharmacol. Exp. Therapeutics.*, **252**, 979.

RASMUSSEN, H. & BARRETT, P.Q. (1984). Calcium messenger system: an integrated view. *Physiol. Rev.*, **64**, 938.

REEVE, J.G., RABBITS, P.H. & TWENTYMAN, P.R. (1990). Non-P-glycoprotein-mediated multidrug resistance with reduced EGF receptor expression in a human large cell lung cancer cell line. *Br. J. Cancer*, **61**, 851.

ROTMAN, B. & PAPERMASTER, B.W. (1966). Membrane properties of living mammalian cells studied by enzymatic hydrolysis of fluorogenic esters. *J. Immunol. Methods.*, **55**, 124.

SAFA, A.R., GLOVER, C.J., SEWELL, J.L., MEYERS, M.B., BIEDLER, J.L. & FELSTED, R.L. (1987). Identification of the multidrug resistance-related membrane glycoprotein as an acceptor for calcium channel blockers. *J. Biol. Chem.*, **262**, 7884.

SCHEPER, R.J., BULTE, J.W.M., BRAKKEE, J.G.P. & 8 others (1988). Monoclonal antibody JSB-1 detects a highly concerved epitope on the glycoprotein associated with multi-drug-resistance. *Int. J. Cancer*, **42**, 389.

SIMPSON, W.G. (19850. The calcium channel blocker verapamil and cancer chemotherapy. *Cell Calcium*, **6**, 449.

SLATER, L.M., SWEET, P., STUPECKY, M. & GUPTA, S. (1986). Cyclosporin A reverses vincristine and daunorubicin resistance in acute lymphatic leukemia *in vitro*. *J. Clin. Invest.*, **77**, 1405.

STEEN, A.M., LUTHMAN, H., HELLGREN, D. & LAMBERT, B. (1990). Levels of hypoxanthine phosphoribosyltransferase RNA in human cells. *Exp. Cell. Res.*, **186**, 236.

TSIEN, R.Y., POZZAN, T. & RINK, T.J. (1982). Calcium homeostasis in intact lymphocytes: cytoplasmic free calcium monitored with a new, intracellularly trapped fluorescent indicator. *J. Cell. Biol.*, **94**, 325.

TSURUO, T., IIDA, H., KAWABATA, H., TSUKAGOSHI, S. & SAKURAI, Y. (1984). High calcium content of pleiotropic drug-resistant P388 and K562 leukemia and Chinese hamster ovary cells. *Cancer Res.*, **44**, 5095.

UEDA, K., CLARK, D.P., CHEN, C., RONINSON, I.B., GOTTESMAN, M.M. & PASTAN, I. (1987). The human multidrug resistance (*mdr*1) gene. cDNA cloning and transcription initiation. *J. Biol. Chem.*, **262**, 505.

VAYUVEGULA, B., SLATER, L., MEADOR, J. & GUPTA, S. (1988). Correction of altered plasma membrane potentials: a posible mechanism of cyclosporin A and verapamil reversal of pleiotropic drug resistant in neoplasia. *Cancer Chemother. Pharmacol.*, **22**, 163.

YUSA, K. & TSURUO, T. (1989). Reversal mechanism of multidrug resistance by verapamil: direct binding of verapamil to P-glycoprotein on specific sites and transport of verapamil outward across the plasma membrane of K562/ADM cells. *Cancer Res.*, **49**, 5002.